# A 23-Plex Cytokine/Chemokine Analysis Identifies TNFRII, MMP-8, and sIL-1RII as Potential Biomarkers for Systemic Sclerosis

**DOI:** 10.3390/biomedicines13040967

**Published:** 2025-04-16

**Authors:** Carlo Perricone, Giacomo Cafaro, Roberto dal Pozzolo, Lorenza Bruno, Nicole Sasso, Roberta Cecchetti, Matteo Antonucci, Fabiana Topini, Onelia Bistoni, Patrizia Mecocci, Roberto Gerli, Elena Bartoloni

**Affiliations:** 1 Section of Rheumatology, Department of Medicine and Surgery, University of Perugia, 06129 Perugia, Italy; giacomo.cafaro@unipg.it (G.C.); roberto.dalpozzolo@gmail.com (R.d.P.); lorenza.bruno@ospedale.perugia.it (L.B.); nicole.sasso@gmail.com (N.S.); matteo.antonucci@virgilio.it (M.A.); fabiana.topini@ospedale.perugia.it (F.T.); onelia.bistoni@oseodale.perugia.it (O.B.); roberto.gerli@unipg.it (R.G.);; 2 Section of Gerontology and Geriatrics, Department of Medicine and Surgery, University of Perugia, 06129 Perugia, Italy; roberta.cecchetti@unipg.it (R.C.); patrizia.mecocci@unipg.it (P.M.); 3 Division of Clinical Geriatrics, NVS Department, Karolinska Institute, 17177 Stockholm, Sweden

**Keywords:** systemic sclerosis, biomarkers, cytokines, TNFRII, MMP-8, IL-1RII

## Abstract

**Background:** Reliable biomarkers are urgently needed to aid in the differential diagnosis, prognosis, disease progression monitoring, and prediction of therapeutic response in patients with systemic sclerosis (SSc). This study aimed to evaluate a panel of potentially pathogenic circulating cytokines and chemokines in a cohort of SSc patients. **Methods:** Serum samples were obtained from 35 SSc patients and 40 age- and sex-matched healthy controls. Twenty-three cytokines/chemokines were quantified using a Luminex^®^ multiplex immunoassay (BioRad-BioPlex 200 System-Lumine x-Map technology R&D Systems, USA) following the manufacturer’s instructions and customized procedures. Data were acquired using Bioplex manager v 6.1. Data analysis was performed using GraphPad Prism v.8 (GraphPad Software, Inc.), with significance defined as *p* ≤ 0.05. V.8 **Results:** Levels of TNFRII and MMP-8 were significantly higher in SSc patients compared to healthy controls, while IL-1RII levels were significantly elevated in healthy individuals (*p* < 0.001 for all comparisons). Patients with elevated ESR at baseline (>30 mm/h) showed higher IL-15 levels (*p* = 0.019). A strong positive correlation was found between MCP-1 and the modified Rodnan skin score (mRSS) (*p* = 0.009, r = 0.740), and a moderate correlation between TNFRII and diffusing capacity for carbon monoxide (*p* = 0.046, r = 0.339). PLS regression identified MMP-8, MCP-1, TNFRII, IL-15, and IL-1RII as key predictors of SSc, with MMP-8 having the strongest influence. The logistic regression model showed high performance (AUC = 0.93, accuracy = 87.5%). **Conclusions:** TNFRII, MMP-8, and IL-1RII may play a pathogenic role in SSc. TNFRII, in particular, may serve as a biomarker for pulmonary involvement, aligning with its known role in pro-fibrotic pathways. These findings support their utility in diagnosis and disease profiling.

## 1. Introduction

Systemic sclerosis (SSc) is a complex systemic autoimmune disease characterized by microvascular damage and excessive fibrosis of skin and internal organs, including heart, lungs, kidneys, and gastrointestinal tract [1]. One of the major hallmarks of the disease is the abnormal production of collagen and extracellular matrix, which causes fibrosis in the affected tissues.

Immunologic and vascular processes are believed to be central to the pathogenesis of SSc. However, the initial triggering events that facilitate the development of the pathomechanisms involved in SSc are still unclear. SSc is usually classified into two distinct clinical subtypes: limited cutaneous SSc and diffuse cutaneous SSc. This distinction is based on the extent of skin involvement. lcSSc is defined as skin thickening distal to the elbows and knees, with or without involvement of the face. dcSSc is instead characterized by the presence of skin thickening extending to arms and thighs, with or without the involvement of the face. Measurement of skin thickness is used as a surrogate for disease activity and severity in patients with scleroderma. The modified Rodnan skin score (mRSS) is a measure of skin thickness and is used as a primary or secondary outcome measure in clinical trials of systemic sclerosis [2].

The two subtypes of SSc are also distinguished by distinct serological profiles, clinical features, and organ involvement. One of the main complications related to SSc is lung disease. This can be due to interstitial lung disease or pulmonary arterial hypertension [3]. Pulmonary function tests are an important tool in the initial screening of patients with SSc. These tests can detect alterations before respiratory symptoms appear.

The reduction in diffusing capacity for carbon monoxide (DLCO) is the main detectable functional change [4]. DLCO is a measure of the lung’s capacity for gas exchange. It depends on the membrane diffusing capacity and pulmonary capillary volume, as well as the red cell resistance. Any pathological condition that causes loss or destruction of the pulmonary capillary membranes (e.g., fibrosis, pulmonary hypertension, emphysema) can alter DLCO. Patients with decreased DLCO (values lower than 40% of predicted) are known to have a poor outcome [5].

Patients with SSc have different clinical features, disease progression, therapeutic response, and prognosis [6]. Except for autoantibodies, no other biomarkers specific to SSc and adopted in clinical practice have been identified yet, significantly limiting the ability to stratify patients early, in order to improve and tailor their management.

Many potentially relevant cytokines or growth factors may be important in the pathogenesis or might be useful markers of pathobiology in SSc. In the present study, we investigated the serum levels of a specific panel of cytokines and chemokines potentially involved in the pathogenesis of SSc compared with healthy subjects.

## 2. Materials and Methods

### 2.1. Patients

The study was conducted in accordance with the principles embodied in the Declaration of Helsinki. Approval was provided by the Ethics Committee “Comitato Etico Regionale Umbria” (study number 4299/2022; approval date on 27 April 2022, protocol of approval number 24418/22/OV). We recruited 35 consecutive patients with systemic sclerosis according to the ACR/EULAR classification criteria. Forty healthy subjects were enrolled in the age- and gender-matched control group.

The clinical variables recorded were gender, age at diagnosis, cigarette smoking habit, body mass index, disease duration, Raynaud’s phenomenon, presence of ulcers, mRSS, joint, cardiac, pulmonary, gastrointestinal, and hematological involvement (anemia, leukopenia, and thrombocytopenia), arterial hypertension, systolic pulmonary artery pressure (SPAP) value (mmHg) and DLCO (%) [7,8]. Disease-specific laboratory parameters included ANA (by indirect immunofluorescence, normal values < 1:80), anti-Scl70, anti-centromere, anti-SSA, anti-SSB (by immunoblotting, normal values < 15 U/L), rheumatoid factor (normal value < 14 U/L), complement levels C3 and C4 (by nephelometric method, normal values 90–180 mg/dL and 15–40 mg/dL, respectively), ESR (normal value < 30 mm/h), and CRP values (normal value < 0.5 mg/dL) at disease onset and gammaglobulinaemia. Each parameter was defined according to its laboratory cut-off. Current and previous therapies, including glucocorticoids (prednisone ≥ 7.5 mg/daily or equivalent), immunosuppressants (such as azathioprine, cyclophosphamide, cyclosporine A, mycophenolate mofetil, methotrexate, leflunomide, and rituximab), and hydroxychloroquine were recorded.

### 2.2. Cytokine Profile and Analysis

Blood samples in heparin tubes were collected and centrifuged at the end of the clotting time (30–60 min) for 15 min ~2000× *g* at room temperature. The aliquots of serum were stored at −80 °C.

Samples were thawed at room temperature before the analysis. Twenty-three cytokines/chemokines (CCL2/MCP-1, IP-10/CXCL10, Flt3 Ligand, IFNa2, IL6, IL7, IL12, IL13, IL15, IL17A, TNFa, IL-1Ra, CXCL13, IL21, IL23, IL33, TSLP, IL22Ra, IL1RII, TNFRII, BAFF, CCL19/MIP-3B, MMP-8) were quantified using Luminex^®^ multiplex immunoassay (BioRad- BioPlex 200 System—Lumine x-Map technology R&D Systems, Minneapolis, MN 55413, USA) following the manufacturer’s instructions and customized procedures. Briefly, the aliquots were diluted to a ratio of 1:4 and assayed in 96-well plates. The content of each well was then pumped into the Bio-Plex 200 System array reader, in order to quantify each specific reaction based on the color of the beads and the intensity of the fluorescent signal. Data were acquired using Bioplex manager v 6.1.

### 2.3. Statistical Analysis

To assess data distribution, we used the D’Agostino–Pearson omnibus test, followed by the application of the most appropriate parametric or non-parametric tests based on the resulting distribution characteristics. An initial univariate analysis was conducted for exploratory purposes, followed by multivariate analysis to evaluate associations among study variables. A multiple logistic regression analysis was performed to identify independent predictors of systemic sclerosis (SSc). The model demonstrated good discriminatory ability, with an overall classification accuracy of 87.5% and an area under the receiver operating characteristic (ROC) curve (AUC) of 0.93, indicating excellent performance. Additionally, Partial Least Squares (PLS) Regression was employed to model the relationship between cytokine expression profiles and the risk of SSc, using the implementation provided by the scikit-learn library [9]. This approach was chosen due to its ability to handle multicollinearity and reduce dimensionality in datasets with highly correlated variables. Lasso regularization was applied to improve model stability, shrink irrelevant coefficients, and enhance interpretability. The PLS model achieved a Mean Squared Error (MSE) of 0.632, reflecting a moderate level of prediction error. For group comparisons, either the Mann–Whitney U test or Student’s *t*-test were used, depending on data normality. Correlation analyses were performed using Pearson or Spearman’s correlation coefficients, as appropriate. A two-tailed *p*-value of < 0.05 was considered statistically significant. All statistical analyses were performed using GraphPad Prism version 8.0 (GraphPad Software, Inc., La Jolla, CA, USA) and IBM SPSS Statistics version 20.0 (IBM Corp., Armonk, NY, USA).

## 3. Results

We evaluated a panel of 23 cytokines and chemokines in a group of SSc patients (n = 35) and compared the results with those obtained from a population of healthy subjects (n = 40). The clinical and demographic characteristics of these subgroups are shown in Table 1, while the laboratory features and the treatment used by SSc patients are reported in Table 2. Among the SSc patients, 33 (94.3%) were female and 2 (5.7%) were male. Thirteen SSc patients (37.4%) were current (10, 28.6%) or former smokers (3, 8.6%). The mean age was 60.5 ± 13.8 years (mean ± SD) with a mean disease duration of 95.5 ± 97.22 months (mean ± SD). Thirty-four (97.1%) patients presented with Raynaud’s phenomenon, and among them 17.1% had a history of digital ulcers. Skin involvement was present in 27 (77.1%) patients with a mean mRSS of 5.5 ± 5.8 (mean ± SD). Eight patients (22.8%) had joint involvement, while none had cardiac involvement. Fifteen patients (42.7%) had a pulmonary involvement with interstitial lung disease with a mean DLCO of 78 ± 22%. Pulmonary hypertension was found in 10/35 (28.6%) with a mean value of SPAP of 33.5 ± 8.3 mmHg. Gastrointestinal involvement occurred in seven (20%) patients. Finally, among all the patients studied, 3 (8.6%) were obese (BMI > 30) and 11 (31.4%) had hypertension. The control population consisted of 40 healthy, age-matched female subjects with a mean age of 57 ± 12.8 years (mean ± SD).

**Figure 1:**
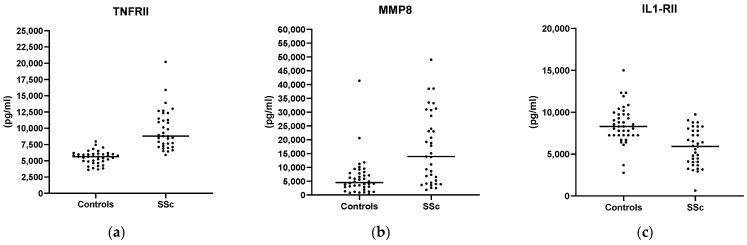
Differences in TNFRII (**a**), MMP-8 (**b**), and IL-1RII (**c**) levels between healthy controls and SSc patients (*p* < 0.001 for all comparisons).

**Figure 2:**
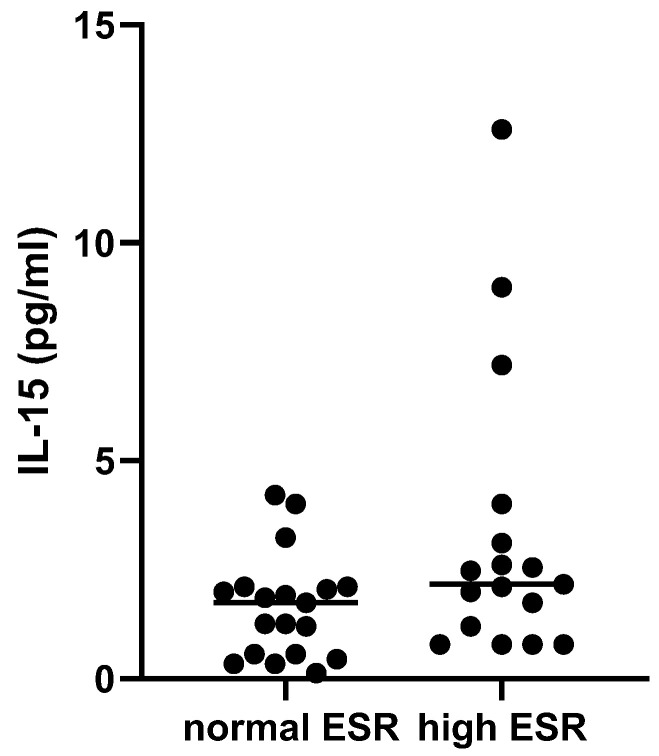
IL-15 levels in patients with normal and increased ESR at baseline (≤30 mm/h, vs. >30 mm/h) (*p* = 0.019).

**Figure 3:**
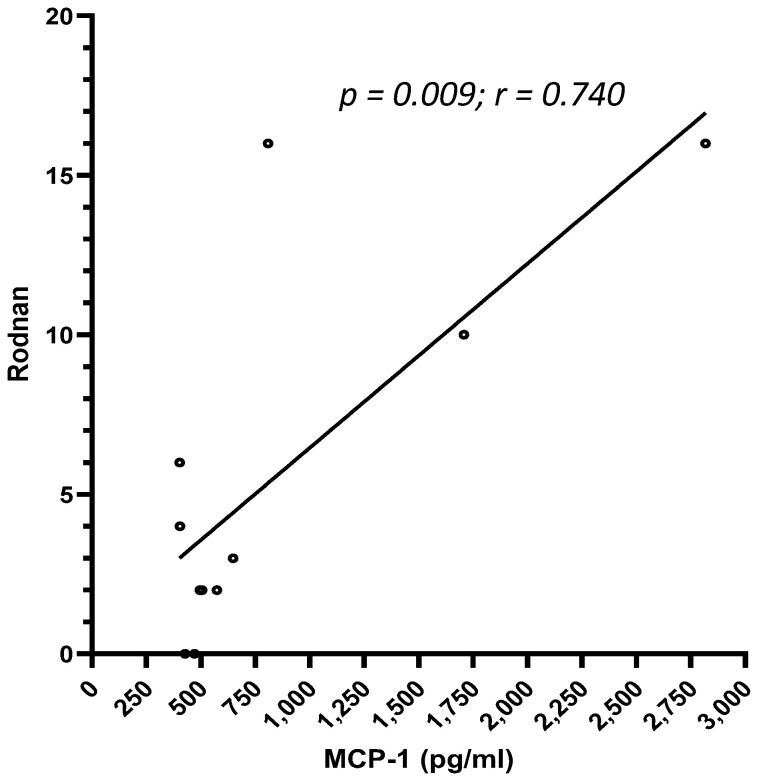
Correlation between MCP-1 levels and mRSS (*p* = 0.009; r = 0.740).

**Figure 4:**
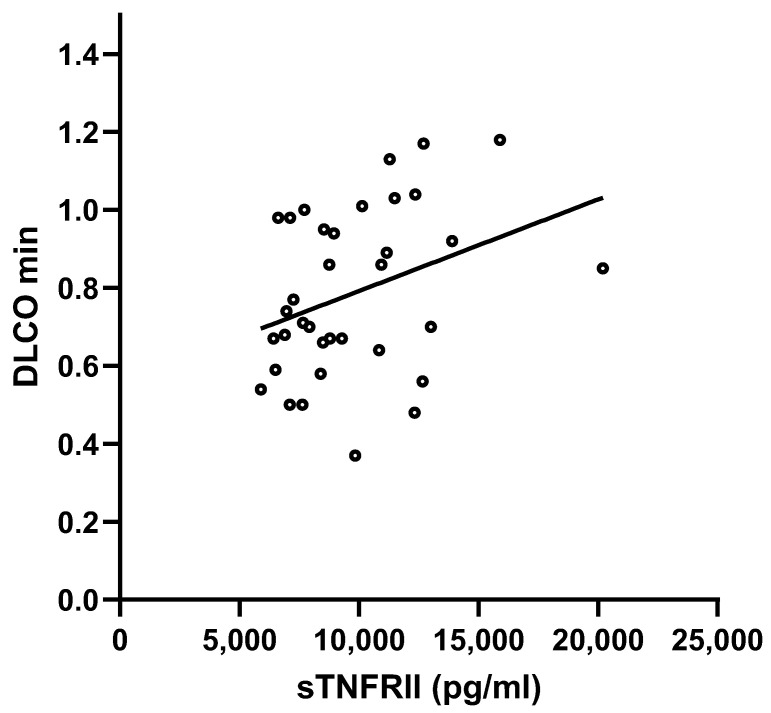
Correlation between TNFRII and DLCO (*p* = 0.046; r = 0.339).

We compared the cytokines expression of patients with SSc to healthy subjects and found that TNFRII and MMP-8 levels (Figure 1a,b) were higher in SSc patients than in healthy controls (TNFRII 9761 ± 3076 pg/mL vs. 5504 ± 1007 pg/mL and MMP-8 16,839 ± 12,902 pg/mL vs. 6247 ± 6950 pg/mL, respectively), whereas IL-1RII concentration (Figure 1c) was higher in healthy subjects (8514 ± 2255 vs. 5784 ± 2220 pg/mL) (*p* < 0.001 for all comparisons). No further difference among the studied cytokines levels was found (Supplementary Table S1). Patients with increased ESR at baseline (>30 mm/h) presented with higher concentrations of IL-15 (1.7 ± 1.5 vs. 1.2 ± 1.2 pg/mL, *p* = 0.019) (Figure 2). We also observed a positive correlation between MCP-1 and the mRSS (*p* = 0.009; r = 0.740) (Figure 3) and a positive correlation between TNFRII and DLCO (*p* = 0.046; r = 0.339) (Figure 4).

Therefore, we performed a multiple logistic regression analysis (accuracy: 87.5%, area under the ROC curve: 0.93) with risk of SSc as dependent variable and all variants as independent variables.

No variable was independently associated with the risk of SSc (Table 3).

**Figure 5:**
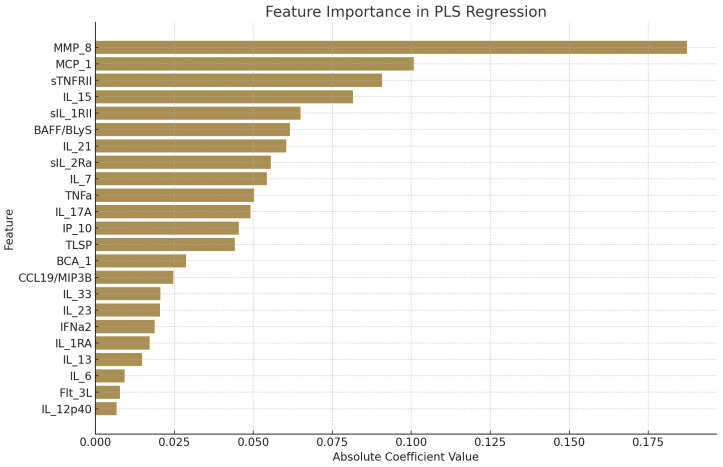
Relative importance of each predictor in the partial least squares regression (PLS) model, measured by the absolute value of their regression coefficients. Higher values indicate a stronger influence on the risk of SSc.

Then, partial least squares regression (PLS regression) was applied to predict the risk of SSc using all the set of 23 cytokines as biomarker features. The trained model had a mean squared error (MSE) of 0.632, indicating a moderate level of prediction error. Score and loading plots are provided in Figure 5.

The PLS regression analysis identified MMP-8, MCP-1, sTNFRII, IL-15, and sIL-1RII as the most influential predictors of SSc diagnosis, with MMP-8 suggesting a strong association, with SSc classification having the highest absolute coefficient (0.187).

**Figure 6:**
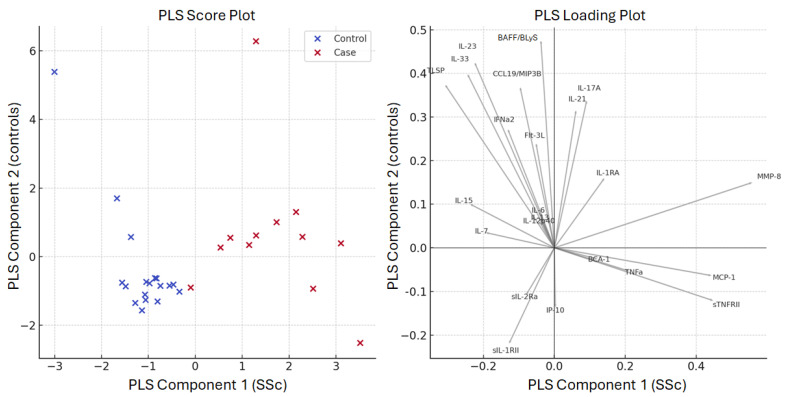
(**Left**) Score Plot: Distribution of the samples along the partial least squares (PLS) regression analysis components. Cases are displayed in red and controls in blue. (**Right**) Loading Plot: Contribution of each biomarker to each PLS component. The distance of the arrows from the center indicates the strength of the influence; the direction reflects the relationship with the component axes (on x cases and on y controls).

Figure 6 displays the most important predictors based on the absolute values of the regression coefficients as a bar chart.

## 4. Conclusions

In this study, we evaluated a panel of cytokines and chemokines potentially involved in the pathogenesis of SSc. Our study aimed to identify possible factors that could predict phenotypic characteristics and could stratify disease severity [10]. We found that TNFRII, MMP-8, and IL-1RII are differently present in patients with SSc compared with healthy subjects, suggesting a potential pathogenic role. Moreover, TNFRII was confirmed to be associated with risk of SSc and could be useful in identifying SSc patients with pulmonary involvement. These results are consistent with a role in pro-fibrotic pathways played by these molecules.

To the best of our knowledge, this is the first study to investigate the role of IL-1RII in SSc. We observed a reduced production of this cytokine in SSc patients compared to healthy controls. The role of this cytokine is still controversial. On the one hand, according to Kawaguchi and collaborators [11], IL-1RII is fundamental in the formation and functioning of the pre-IL-1β complex, which, after stimulation by IL-6, induces the production of procollagen by fibroblasts [12]. On the other hand, the role played by IL-1RII is very intriguing since this cytokine seems to inhibit IL-1 function in synovial and epithelial cells. Cells expressing IL-1RII appear to be more resistant to IL-1β-induced production of NO, PGE2, IL-6, and IL-8 than IL-1rII (−) cells. Furthermore, IL-1RII (+) chondrocytes, when transplanted onto human osteoarthritis-affected cartilage in vitro, appear able to inhibit the spontaneous production of NO and PGE2 in cartilage ex vivo [12]. A reduced production of IL-1RII may not be able to confer significant protection to the cells from the autocrine and paracrine effects of IL-1, thus, exposing them to an increased risk related to the oxidative stress induced by NO, a key phenomenon in the pathogenesis of SSc. In addition to that, previous studies demonstrated the regulatory role of decoy IL-1RII on IL-1β-mediated IL-1R signaling affecting Th17 responses [13]. Given the role of these cells in the pathogenesis of SSc, it cannot be excluded that the reduced production of IL-1RII in patients with SSc may promote the pro-fibrotic activity of Th17 cells.

Moreover, we explored the potential role of MMP8 as a biomarker for SSc for the first time. MMP8 is a potent interstitial collagenase believed to be mainly expressed by polymorphonuclear neutrophils. In their study, Craig et al. observed increased levels of MMP-8 mRNA and protein in the lungs and bronchoalveolar lavage samples of wild-type mice treated with bleomycin but not in Mmp-8(-/-) mice [14]. Activated murine lung fibroblasts express MMP-8 in vitro. MMP-8 expression is also increased in leukocytes in the lungs of patients with idiopathic pulmonary fibrosis compared to control lung samples. Therefore, it appears that MMP-8 can reduce the acute lung inflammatory response during lung injury but equally promote lung fibrosis. In murine models, the deficiency of MMP8 increases the severity of rheumatoid arthritis, leading to a greater expression of IL-1β [15]. According to our results, MMP8 could be useful in diagnosing patients with SSc.

Interestingly, the present results seem to confirm a potential pathogenic role of TNFRII. In this setting, Tolusso et al. demonstrated the association between a rare TNFRII gene polymorphism (196 T/G) and an increased susceptibility to developing SSc [16]. However, for the lower frequency of the GG genotype in patients and controls, a much larger study population is needed to delineate the role of this polymorphism in patients with SSc. Gruschwitz et al. observed that TNFRII is found on lymphocytes and 30–50% of endothelial cells in early skin SSc lesions [17]. Moreover, they demonstrated that serum levels of soluble TNFRII correlate with their in situ expression and with clinical and laboratory signs of inflammation and disease progression. This may support a potential cytokine role in favoring organ involvement in SSc, as also detected in our cohort, in which cytokine levels correlated positively with greater pulmonary involvement and DLCO levels.

Also, we confirm that patients with SSc may display increased levels of IL-15 [18]. This pleiotropic cytokine is characterized by various effects on immune, vascular, and connective tissue cells. It was shown that higher IL-15 production was associated with high SPAP levels and pulmonary fibrosis [18]. The impact of IL-15 on the development of fibrosis is still not fully understood [19,20]. However, due to its association with decreased lung function, IL-15 could potentially serve as a useful biomarker for lung involvement in SSc and a potential target for therapy.

Furthermore, we provided a possible correlation between monocyte chemotactic protein-1 (MCP-1) levels and the mRSS. MCP-1 is a potent chemokine which is upregulated in fibroblasts during the development of sclerosis [21]. In particular, it has been demonstrated that the −2518 promotor polymorphism in the MCP-1 gene is associated with SSc [21]. Hasegawa and colleagues were the first to study the involvement of MCP-1 in SSc [22]. They demonstrated that MCP-1 levels were significantly higher in PBMCs and serum of SSc patients compared to healthy controls and seemed to correlate with pulmonary fibrosis. MCP-1 was also found to be overexpressed in the lesional skin of scleroderma. While the association of MCP-1 with pulmonary fibrosis remains controversial, subsequent studies have confirmed the role of MCP-1 in skin fibrosis, as confirmed in our study. [23,24].

SSc is a rare inflammatory disease in which biomarkers are still frequently lacking. We raise the possible utility of dosing MMP8 for SSc diagnosis. Furthermore, we suggest a potential pathogenic role for IL-RII, and we confirm that IL-15, TNFRII, and MCP-1 may be involved in pathogenesis and characterize the disease phenotype.

## Figures and Tables

**Table 1:** Clinical and demographic characteristics of patients with systemic sclerosis (SSc) compared with healthy controls.

SSc (n = 35)		Controls (n = 40)	
Sex (F/M)	33 (94.3%)/2 (5.7%)	40 F (100%)	*p* = ns
Age (years, mean ± SD)	60.5 ± 13.8	57 ± 12.8	*p* = ns
Disease duration (months, mean ± SD)	96.5 ± 97.22		
Smoking	13 (37.4%)		
Raynaud’s phenomenon	34 (97.1%)		
Skin involvement	27 (77.1%)		
Digital ulcers	6 (17.1%)		
mRSS (mean ± SD)	5.5 ± 5.8		
Joint involvement	8 (22.8%)		
Cardiac involvement	0		
sPAP (mmHg, mean ± SD)	30.5 ± 8.3		
Skin involvement	15 (42.7%)		
DLCO (%, mean ± SD)	78 ± 22		
Gastrointestinal involvement	7 (20%)		

mRSS: modified Rodnan skin score; sPAP: systolic pulmonary artery pressure; DLCO: diffusing capacity of the lungs for carbon monoxide.

**Table 2:** Laboratory features and treatment of systemic sclerosis (SSc) patients.

SSc Patients (n = 35)	
ANA	34 (97.1%)
Rheumatoid factor	7 (20%)
Anti-Scl70 (U/mL)	29.3 ± 61.2
Anti-CENP B (U/mL)	55.9 ± 47.3
Anti-SSA	7 (20%)
Anti-SSB	1 (2.8%)
Low C3	1 (2.8%)
Low C4	1 (2.8%)
Elevated ESR	7 (20%)
Elevated CRP	5 (14.3%)
Anemia	7 (20%)
Leukopenia	3 (8.6%)
Thrombocytopenia	0
Hypergammaglobulinemia	4 (11.4%)
Dose of glucocorticoids (equivalent dose of prednisone, mg/day)	2.2 ± 5.4
Current DMARDs therapy	12 (34.3%)
HCQ	11 (31.4%)
MTX	10 (28.6%)
AZA	2 (5.7%)
CyA	1 (2.8%)
MMF	3 (8.6%)
Iloprost	6 (17.1%)
Calcium channel blockers (nifedipine/amlodipine)	12 (34.3%)

ANA: anti-nuclear antibodies; DMARDs: disease-modifying anti-rheumatic drugs; HCQ: hydroxychloroquine; MTX: methotrexate; AZA: azathioprine; CyA: cyclosporin-A; MMF: mycophenolate mofetil.

**Table 3:** Final model of multiple logistic regression analysis with reported beta estimates of SSc diagnosis.

Variable	Beta Estimate	Lower 95% CI	Upper 95% CI	*p*-Value
MMP-8	1.466	0.859	2.073	0.508
MCP-1	0.728	0.142	1.314	
IL-21	0.501	−0.007	1.009	0.907
sTNFRII	0.468	0.021	0.914	
IFNa2	0.326	−0.211	0.864	
IL-13	0.251	−0.337	0.838	0.912
BAFF/BLyS	0.222	−0.836	1.281	
TNFa	0.221	−0.782	1.223	
BCA-1	0.144	−0.801	1.088	
IL-17A	0.139	−0.419	0.698	
CCL19/MIP3B	0.057	−0.411	0.526	0.958
IL-6	0.053	−0.549	0.654	
Flt-3L	0.021	−0.734	0.775	
IL-12p40	−0.078	−0.811	0.655	0.982
IL-33	−0.238	−2.302	1.825	
IL-1RA	−0.261	−0.750	0.228	
sIL-1RII	−0.290	−0.589	0.008	
IL-23	−0.327	−2.215	1.561	0.868
IP-10	−0.380	−0.785	0.025	
sIL-2Ra	−0.396	−1.074	0.281	0.953
TLSP	−0.400	−1.311	0.511	
IL-7	−0.404	−0.802	−0.005	1.000
IL-15	−0.697	−1.431	0.038	0.922

## Data Availability

Data and materials are available from the corresponding author upon request.

## Supplementary Materials

The following supporting information can be downloaded at https://www.mdpi.com/article/10.3390/biomedicines13040967/s1: Table S1: levels of studied cytokines in SSc patients and controls.

## List of Abbreviations

SSc	Systemic sclerosis
CCL2/MCP-1	Monocyte chemotactic protein 1
IP-10/CXCL10	Interferon (IFN)-γ-induced protein-10
Flt3 Ligand	FMS-like tyrosine kinase 3
IFNa2	Interferon Alpha 2
IL	Interleukin
TNFa	Tumor necrosis factor alpha
CXCL13	CXC motif chemokine ligand 13
TSLP	Thymic stromal lymphopoietin
TNFRII	Tumor necrosis factor-RII
BAFF	B-cell activating factor
CCL19/MIP-3B	Macrophage Inflammatory Protein-3-beta
MMP-8	Matrix metalloproteinase-8
mRSS	Modified Rodnan skin score
DLCO	diffusing capacity for carbon monoxide
SPAS	Systolic pulmonary artery pressure
ANA	Antinuclear antibodies
ESR	Rrythrocytes sedimentation rate
CRP	C-reactive protein
BMI	Body mass index
DMARDs	Disease-modifying anti-rheumatic drugs
HCQ	Hydroxychloroquine
MTX	Methotrexate
AZA	Azathioprine
CyA	Cyclosporin-A
MMF	Mycophenolate mofetil
