# Supplementary material for: A 23-Plex Cytokine/Chemokine Analysis Identifies TNFRII, MMP-8, and sIL-1RII as Potential Biomarkers for Systemic Sclerosis
Source: Biomedicines. 2025 Apr 16;13(4):967. doi: 10.3390/biomedicines13040967 (PMC12025288; doi:10.3390/biomedicines13040967)
Supplement: Supplementary file 1 [file biomedicines-13-00967-s001.zip › biomedicines-3434781-supplementary.pdf]

Supplementary Table S1. Cytokines panel analysis in SSc patients and healthy controls.

Cytokines	SSc (n=35) (mean ± SD, pg/ml)	Healthy controls (n=40) (mean ± SD, pg/ml)	
**sIL2Ra**	607.7 ± 294	757.5 ± 370.3	P=ns
**Flt3L**	13.29 ± 9.678	14.31 ± 11.74	P=ns
**IFNa2**	11.66 ± 11.03	18.78 ± 17.77	P=ns
**IL12p40**	4.563 ± 3.69	15.44 ± 24.74	P=ns
**IL13**	15.08 ± 16.22	11.79 ± 30.51	P=ns
**IL17A**	8.736 ± 11.82	2.78 ± 3.416	P=ns
**IL1RA**	44.89 ± 39.97	42.86 ± 33.96	P=ns
**IL6**	11.24 ± 12.86	9.057 ± 23.22	P=ns
**IL7**	1.443 ± 0.9282	2.641 ± 2.272	P=ns
**IP10**	491.7 ± 452.1	599.3 ± 491.5	P=ns
**TNFa**	11.38 ± 15.45	7.98 ± 3.48	P=ns
**CXCL13**	88.86 ± 219.7	66.68 ± 90.55	P=ns
**TLSP**	33 ± 73.94	210.4 ± 315.5	P=ns
**IL33**	184 ± 377.4	687.1 ± 1307	P=ns
**IL21**	17.51 ± 14.41	10.3 ± 13.36	P=ns
**IL23**	4360 ± 10134	13032 ± 21380	P=ns
**CCL19-MIP3B**	599.8 ± 489.4	685.3 ± 702.7	P=ns
**BAFF**	2.964 ± 2.815	3.89 ± 9.43	P=ns
**IL-15**	5.204 ± 14.04	1.368 ± 1.259	P=ns
**MCP-1**	745.5 ± 510.2	654.8 ± 608.9	P=ns
